# Non‐Surgical Periodontal Therapy Modulates Oral Microbiome in Primary Immunodeficient Children

**DOI:** 10.1111/jcpe.14201

**Published:** 2025-07-20

**Authors:** Abish S. Stephen, Stacy Worrall, Cheryl Somani, Robert P. Allaker, Janet Davies, Luigi Nibali, Nikolaos Donos

**Affiliations:** ^1^ Centre for Oral Immunobiology & Regenerative Medicine, Institute of Dentistry, Faculty of Medicine and Dentistry Queen Mary University London London UK; ^2^ Centre for Oral Clinical Research, Institute of Dentistry, Faculty of Medicine and Dentistry Queen Mary University London London UK; ^3^ Department of Paediatric Dentistry The Royal London Hospital, Barts Health NHS Trust London UK; ^4^ Periodontology Unit, Centre for Host Microbiome Interactions, Faculty of Dentistry, Oral & Craniofacial Sciences King's College London London UK

**Keywords:** 16S ribosomal RNA sequencing, microbiota, neutropenia, oral microbiome, periodontal therapy, primary immunodeficiency diseases, subgingival plaque

## Abstract

**Background and Aim:**

Primary Immunodeficiencies (PIDs) arise from rare genetic defects affecting humoral and cellular immunity, which can lead to reduced dental plaque control. This study aimed to characterise the subgingival dental plaque microbiome in neutropenic PID children compared to healthy controls and assess their response to non‐surgical periodontal therapy.

**Methods:**

Subgingival plaque was collected from three first molars and one first incisor at baseline and 6 months post therapy from children with PID (*n* = 24) and systematically healthy control participants (*n* = 24) who were recruited from Great Ormond Street Hospital and Barts Health NHS Trust, respectively. The subgingival microbiome was profiled using an Illumina metabarcoding approach on the bacterial 16S rRNA gene V1–V2 region.

**Results:**

Significant shifts in community structure were observed post therapy, as measured by alpha and beta diversities. An increase in *Rothia* spp., *Neisseria* spp. and *Actinomyces* spp. was noted in PID children post therapy, consistent with clinical improvements. Baseline blood absolute neutrophil counts in PID children were positively associated with *Streptococcus cristatus* and *Gemella* spp., and negatively with *Saccharibacteria, Capnocytophaga* and *Porphyromonas* spp., highlighting key host–microbial relationships.

**Conclusion:**

Non‐surgical periodontal therapy modulated the subgingival microbiota in neutropenic PID children, revealing novel host–microbial interactions important for the oral microbiome in health.

## 1 Introduction

Primary immunodeficiencies (PIDs) encompass a diverse group of conditions characterised by a compromised immune response, making individuals more susceptible to infections, autoimmunity and malignancies. These diseases are grouped according to the component of the immune system that is primarily disrupted: either the adaptive or innate immune system (McCusker and Warrington 2011). Diagnosed in childhood, where 1 in 2000 children under the age of 18 are thought to have a PID, the average interval from onset of symptoms to diagnosis is 2.7 years (Reust 2013). The overall incidence of PID is 1:10,000, with the current number of patients diagnosed in the United Kingdom standing between 4000 and 5000. Within this registry, 4% were congenital defects and the overall percentage of males to females was almost 50/50 (Shillitoe et al. 2018).

These conditions arise from genetic mutations in utero, resulting in either loss of function (LOF) or gain of function (GOF) of a specific encoded protein. This leads to abnormalities in the immune system, as these proteins play a critical role in the development, maintenance and function of immune cells. There are around 485 PIDs, now termed ‘inborn errors of immunity’, including 55 novel monogenic gene defects (Tangye et al. 2022). These disorders, while predominantly affect the body's ability to defend against pathogens, can also profoundly impact oral health, creating unique challenges in dental management (Peacock et al. 2017). PIDs affecting neutrophils are thought to lead to conditions such as aggressive periodontitis, oral ulcers and candidiasis (Atkinson et al. 2000). The interplay between PIDs and these oral health conditions remains complex and not entirely understood, highlighting the need for continuous research.

A notable feature of PIDs is the high prevalence of periodontal disease, an inflammatory condition affecting the supporting structures of the teeth, often resulting in tooth loss if not managed correctly (Halai et al. 2020). PIDs may predispose patients to more severe periodontal disease due to compromised neutrophil function, impairing the body's ability to control bacterial colonisation in the oral cavity. This is particularly the case in neutrophil disorders such as chronic granulomatous disease (CGD) and leukocyte adhesion deficiency (LAD) (Beltrán‐Bustamante et al. 2023; Movahedi et al. 2007). The early onset and aggressive nature of periodontal disease in PID patients have led to it being considered a potential marker for an undiagnosed immunodeficiency, warranting an immunological evaluation for PID (Meyle and Gonzáles 2001).

Management of oral health in PID patients can be challenging because of the increased susceptibility to infections and complications arising from immunodeficiency and the limited literature on the role of the oral microbiome in plaque‐related diseases in these children. Given the increased prevalence of periodontitis in PID, there is a need to understand the microbial basis of plaque‐related diseases such as gingivitis and periodontitis in this group to better inform treatment and management in dental settings or specialist care. This study builds on our previously published work (Nibali et al. 2021), which detailed the clinical periodontal parameters, treatment protocols and patient cohort characteristics. In that paper, we provided comprehensive clinical data, including the use of clinical attachment loss (CAL) for diagnosing periodontitis in paediatric patients, the range of treatments applied and radiographic bone loss assessments. The present report focuses on further understanding the oral microbiome in these patients following periodontal therapy, utilising the same cohort and clinical framework previously reported. We hypothesised that children with PIDs, specifically those with neutrophil dysfunctions, exhibit a distinct subgingival microbiome compared to healthy controls and that non‐surgical periodontal therapy will result in significant shifts towards a more health‐associated microbial community in these patients, in line with improvements observed in clinical indices.

## 2 Methods

### 2.1 Study Design, Participant Selection and Recruitment

This study follows a previously published report on a cohort of children with PIDs and healthy controls (Nibali et al. 2021). The study was conducted in accordance with the ethical principles of the Declaration of Helsinki and International Conference on Harmonisation for Good Clinical Practice (GCP) (ethics approval reference: 15/LO/1090). Recruitment took place at the Great Ormond Street Hospital (GOSH) and the Paediatric Department at Barts Health NHS Trust from March 2017 to December 2019.

Children aged 4–16 years diagnosed with the following neutrophil‐related PIDs were included: (i) disorders of neutrophil numbers (e.g., cyclic, congenital, X‐linked neutropenia); (ii) disorders of neutrophil function (e.g., leucocyte adhesion defects); and (iii) combined immunodeficiency syndromes (e.g., Wiskott–Aldrich syndrome, hyper IgM syndrome). Participants who were no longer neutropenic due to bone marrow transplant or granulocyte colony‐stimulating factor (G‐CSF) therapy were excluded, except those with persistent neutropenia post treatment. Neutropenia was defined as an absolute neutrophil count (ANC) below 1.5 × 10^9^/L, and severe neutropenia was categorised as an ANC below 0.5 × 10^9^/L. Examiner calibration, study visits and periodontal examinations have been described previously (Nibali et al. 2021).

### 2.2 Definition of Periodontal Disease and Non‐Surgical Therapy

Full‐mouth periodontal examinations were conducted, recording the probing pocket depth (PPD), clinical attachment loss (CAL), bleeding on probing (BOP) and visible plaque using the simplified visible biofilm index. Children with BPE codes ≥ 3 received radiographic evaluation. Children were diagnosed as (i) Healthy—no CAL > 3 mm or CAL > 3 mm in < 2 non‐adjacent teeth, and < 15 sites BOP, (ii) Gingivitis—no CAL and ≥ 15 BOP sites and (iii) Periodontitis—CAL > 3 mm in ≥ 2 non‐adjacent teeth. All PID children received oral hygiene instruction and professional mechanical plaque removal (PMPR) with local anaesthesia as needed, over 1–4 visits, using Gracey curettes and ultrasonic devices. Caries was treated as necessary, in shared care with community dental services.

### 2.3 Sample Collection, Extraction and Sequencing

Subgingival plaque was collected from three first molar and one first incisor sites (one in each quadrant) using a curette. If permanent molars were absent, samples were collected from the second primary molars. The samples were pooled together in reduced transport fluid (RTF) on ice and stored at −80°C for further processing. DNA from the subgingival plaque samples was isolated using the MasterPure Gram Positive DNA extraction kit (further details in Appendix A). The bacterial 16S rRNA gene region V1–V2 in the samples was amplified using V1–V2 (Forward: 5′‐AGAGTTTGATYMTGGCTCAG; Reverse: 5′‐TGCTGCCTCCCGTAGRAGT). The libraries were then multiplexed, barcoded and sequenced using the Miseq V3 600 cycle kit in the 300‐bp paired‐end read method on the Illumina Miseq platform (Illumina, USA). The raw fastq files are deposited in the European Nucleotide Archive (BioProject ID: PRJEB76903).

### 2.4 Sequence Processing and Statistical Analysis

The raw Illumina reads were processed using the Divisive Amplicon Denoising Algorithm 2 (DADA2) pipeline (v1.18) implemented in R (v4.3.1) (Callahan et al. 2016). Quality filtering steps, taxonomy assignment and bioinformatic analyses are detailed in the Appendix A. Sequence data were rarefied to a read depth of 5200 across 1000 iterations. Alpha diversity and beta diversity metrics (Bray–Curtis and Jaccard) were calculated using the R package *vegan* (Oksanen et al. 2024).

## 3 Results

A total of 24 children with PIDs and 24 age‐matched healthy controls were assessed. The PID group included patients with various neutrophil disorders, including autoimmune, severe and congenital neutropenia and other syndromes such as Shwachman–Diamond syndrome, Cohen's syndrome and Fanconi's anaemia (Table S1). The mean age at PID diagnosis was 4.2 years, with an average duration since diagnosis of 5.8 years. The majority exhibited neutrophil counts below the normal range, with specific medical interventions such as G‐CSF and antibiotics being common, as described previously (Nibali et al. 2021). Control patients (*n* = 24) were systemically healthy, except for asthma in two cases. Oral hygiene practices, including toothbrushing frequency and the use of interdental brushes and mouthwash, were similar between the groups.

Oral examinations revealed significantly higher prevalence of periodontitis and gingivitis in PID patients, compared to controls, after adjusting for age, gender and plaque (Nibali et al. 2021). Among the PID children, seven (29.2%) had periodontitis, eight (33.3%) had gingivitis and nine (37.5%) were periodontally healthy. Among controls, only 4 (16.7%) presented with gingivitis and 20 (83.3%) were periodontally healthy. PID children exhibited significantly worse periodontal health indicators: mean BOP was 27.5% versus 6.0% in controls (*p* = 0.001); mean PPD was 2.35 versus 1.87 mm in controls (*p* < 0.001); and mean CAL was 1.56 versus 0.84 mm (*p* < 0.001). The average number of sites with PPD > 4 mm was 2.95 per child in the PID group, while controls exhibited virtually none (*p* = 0.043). Despite comparable plaque levels, BOP remained significantly higher among the PID children: with a visible biofilm index (VBI) score of 2, BOP averaged 24.9% in PID children compared to just 2.1% in controls. Radiographs showed evidence of bone loss in four of the seven PID patients diagnosed with periodontitis. Longitudinal assessment after 6 months post treatment showed significant clinical improvements in the test group, although complete resolution to periodontal health was rare (reported in Nibali et al. 2021; Table S2). Improvements were observed in mean PPD (reduction from 2.35 to 1.96 mm, *p* = 0.011) and CAL (1.56 to 1.07 mm, *p* = 0.015). In analysing the microbiome of the study participants, 54 samples remained after exclusion of those with little or no sequences generated. The final sequence count totalled 1,926,566 quality‐filtered sequences, yielding 4462 amplicon sequence variants (ASVs) that uniquely mapped 367 human microbial taxa.

### 3.1 Effect of Non‐Surgical Therapy on the Subgingival Microbiome of PID Children

**FIGURE 1:**
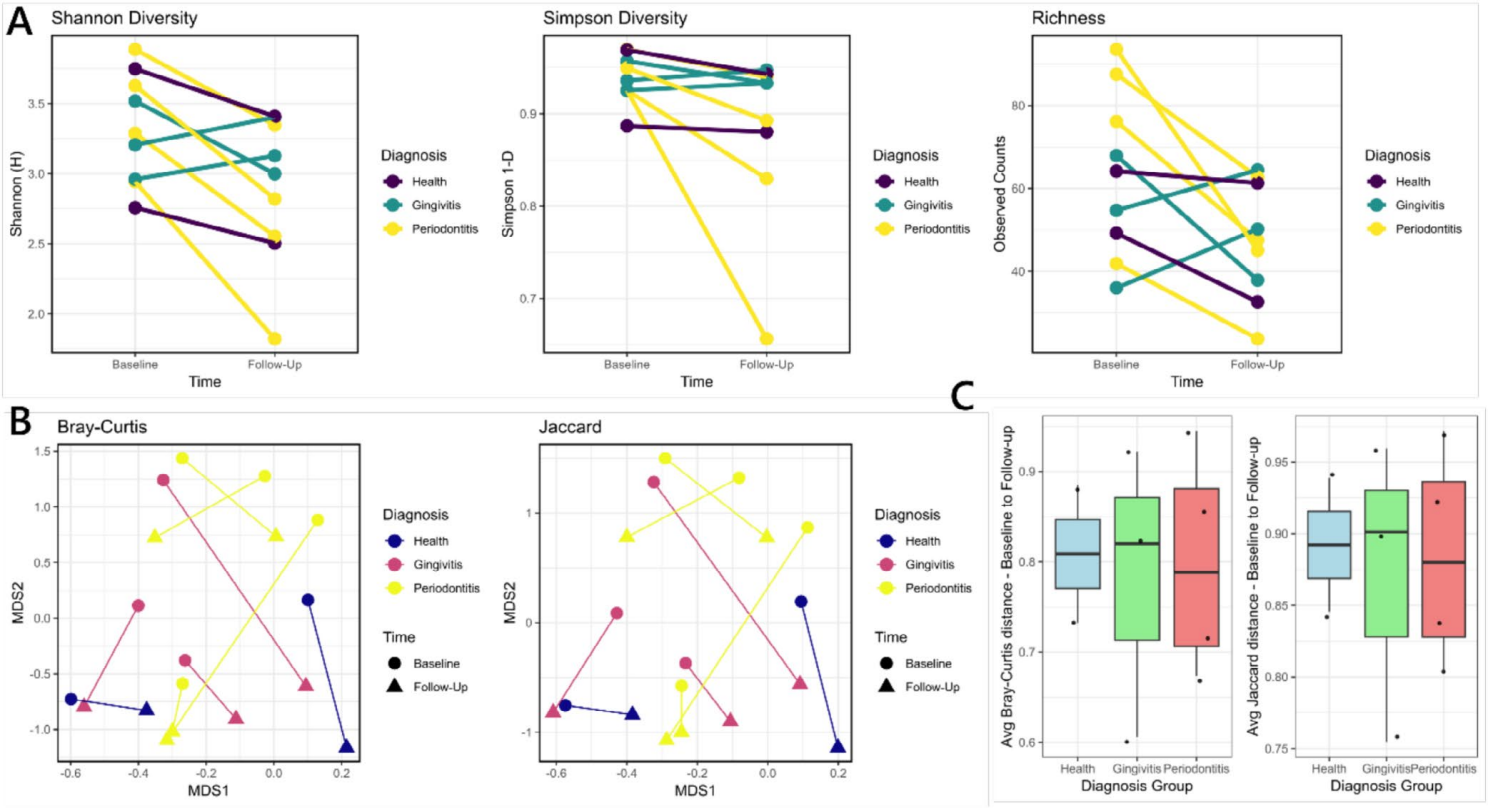
Impact of non‐surgical therapy on microbial diversity and community structure of PID children. (A) Changes in alpha diversity metrics from baseline to 6‐month follow‐up for individual participants. Metrics shown are average Shannon diversity, Simpson diversity and observed richness calculated from 1000 rarefied iterations. (B) Non‐metric multidimensional scaling plots depicting the shifts in rarefied microbial community structure using Bray–Curtis and Jaccard dissimilarity indices. Circles represent baseline samples, and triangles represent follow‐up samples. (C) Boxplots of the average of rarefied dissimilarity between baseline and follow‐up samples using Bray–Curtis (left) and Jaccard (right) indices.

Study participants who underwent PMPR and had follow‐up samples available (*n* = 9) showed marked changes in alpha diversity, with statistically significant reductions in observed richness (*p* < 0.05 in 963 iterations) and Shannon diversity (*p* < 0.05 in 1000 iterations), whereas the Simpson diversity was not significant (Figure 1A). PERMANOVA analysis of the pre‐ and post‐therapy samples in the PID cohort showed significant shifts in the community structure (Bray–Curtis; average *F* = 1.44; *p* < 0.05 in 998 iterations), and the number of shared species (Jaccard; average *F* = 1.23; *p* < 0.05 in 911 iterations; Figure 1B). The average dissimilarity between the pre‐ and post‐therapy samples was > 0.7 for both Bray–Curtis and Jaccard methods (Figure 1C). This group of PIDs included Fanconi's anaemia (FA), severe congenital neutropenia (SCN), autoimmune neutropenia (AN), Papillon–Lefèvre syndrome (PLS), persistent neutropenia (PN) and Kotsmann syndrome (KS), with patients with periodontitis at baseline resolving to health at the 6‐month follow‐up point (Table S3).

### 3.2 Comparison Between the Subgingival Microbiome of PID and Non‐PID Children

**FIGURE 2:**
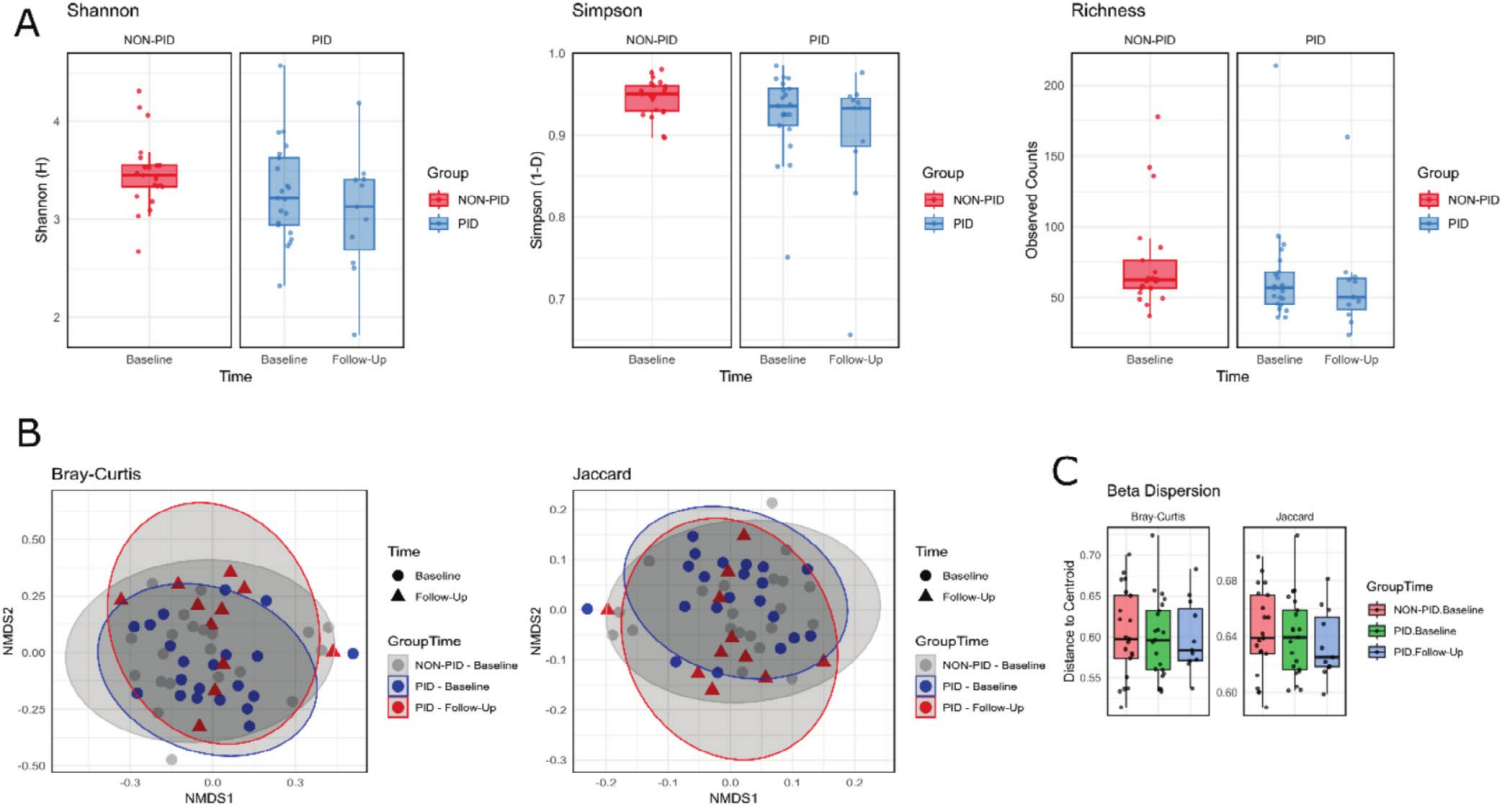
Comparison of alpha and beta diversity between PID and non‐PID samples. (A) Boxplots depicting alpha diversity metrics for Shannon diversity (left), Simpson diversity (middle) and observed richness (right) across different groups and time points. (B) Non‐metric multidimensional scaling plots showing microbial community structure based on Bray–Curtis (left) and Jaccard (right) dissimilarity indices. Points are coloured and shaped by group and time. (C) Boxplots of beta dispersion metrics indicating the distribution of distances to centroids for Bray–Curtis (left) and Jaccard (right) indices across the groups. All metrics shown are mean values of 1000 rarefied iterations for each sample.

In the pooled data, baseline PID samples showed lower alpha diversity than baseline control participant samples, and the PID post‐therapy samples showed lower alpha diversity compared to baseline PID and control samples; however, these comparisons were not statistically significant (Shannon *p* = 0.074; Simpson *p* = 0.15; observed richness *p* = 0.22; Figure 2A).

Analysis of the microbial community structure of PID versus control children along with the post‐therapy samples from the PID cohort showed that the groups are significantly different for both beta diversity measures (Bray–Curtis, *F* = 1.44, *p* < 0.05 in 1000 iterations; Jaccard, *F* = 1.24, *p* < 0.05 in 1000 iterations). Pairwise analysis indicated that baseline sample groups of PID and control were significantly different (adjusted *p* < 0.01 for Bray–Curtis and Jaccard; Figure 2B). Beta dispersion metrics on the rarefied beta diversity analyses indicated that the control group had an overall wider distribution of the distance to centroid values compared to the PID groups (Figure 2C). When stratified into different diagnosis groups, the alpha diversity of the periodontitis group patients was significantly lower at the 6‐month follow‐up compared to the baseline, as opposed to the health and gingivitis groups who showed less drastic reductions in diversity (Figure S1).

### 3.3 Microbial Differences Between PID and Non‐PID Children in the Subgingival Plaque

**FIGURE 3:**
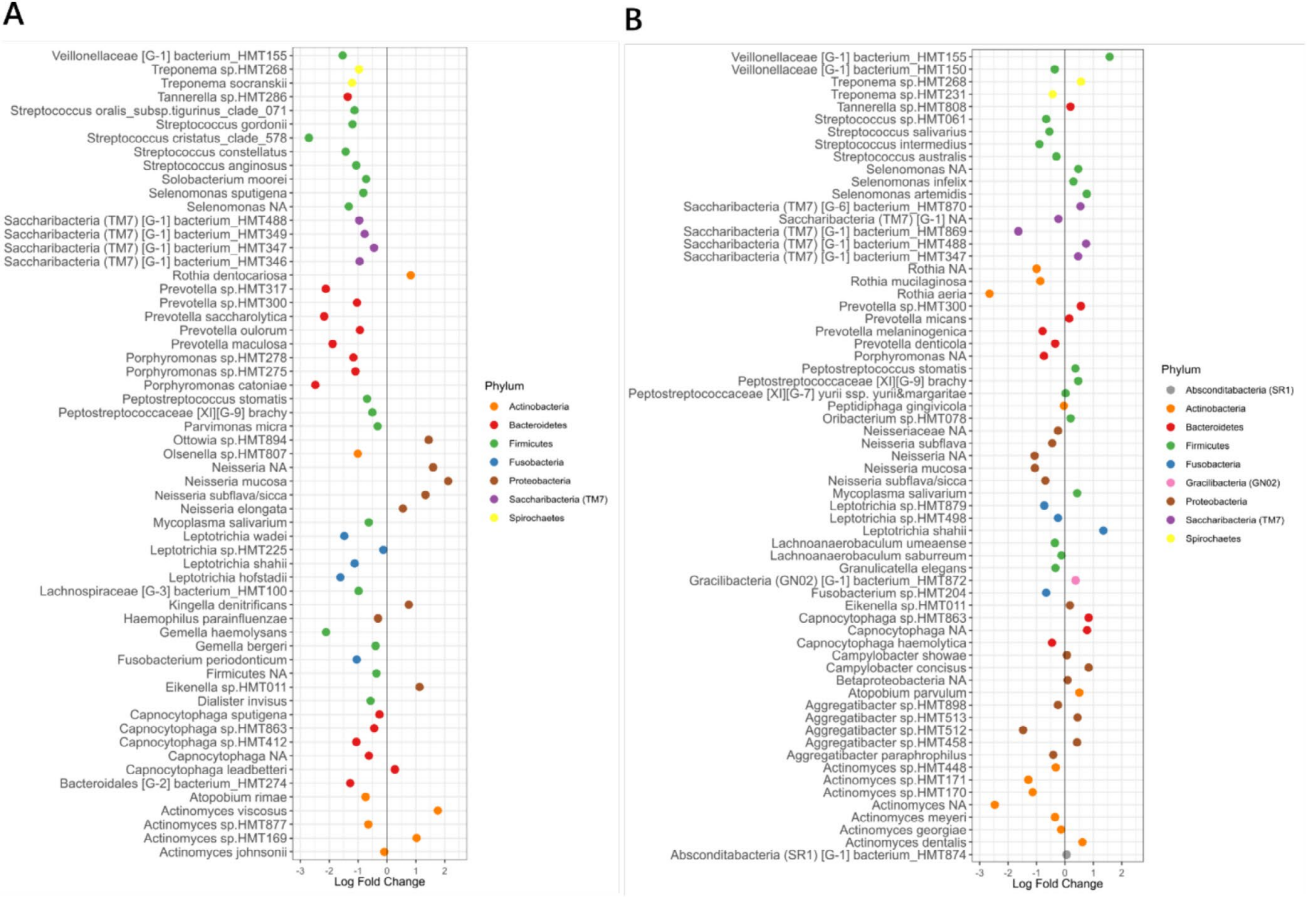
Differential abundance of microbial taxa in PID and non‐PID children using ANCOMBC analysis on non‐rarefied aggregated taxa up to genus and species levels. (A) Log fold changes of all differentially abundant microbial taxa between pre and post therapy in PID children. Positive values indicate higher abundance at follow‐up, and negative values indicate higher abundance at baseline. (B) Log fold changes of all differentially abundant microbial taxa between PID and non‐PID children at baseline. Positive values indicate higher abundance in the PID group, and negative values indicate higher abundance in the non‐PID group. Both plots use the same *x*‐axis scale min‐max limits for comparison of differential abundance patterns.

The analysis of differential abundance of taxa in the subgingival microbiome of PID children before and after therapy detected changes in the abundance of various microbial taxa post therapy. Several key taxa such as *Actinomyces* spp. and members of the Proteobacteria phylum, including *Neisseria mucosa* , *N. elongata* , *N. subflava/sicca*, *Kingella denitrificans, Eikenella* sp. HMT011 and *Rothia dentocariosa* , showed a marked increase in their abundance following therapy (Figure 3A). Certain pathogenic bacteria, including *Bacteriodetes* phylum members such as *Porphyromonas* spp., *Prevotella* spp. and the Spirochaete *Treponema socranskii* showed a decrease in their abundance after the treatment (Figure 3A). A larger decrease in differential abundance was also observed in *Gemella haemolysans* and several species belonging to the *Streptococcus* genus (Figures 3A and S2).

**FIGURE 4:**
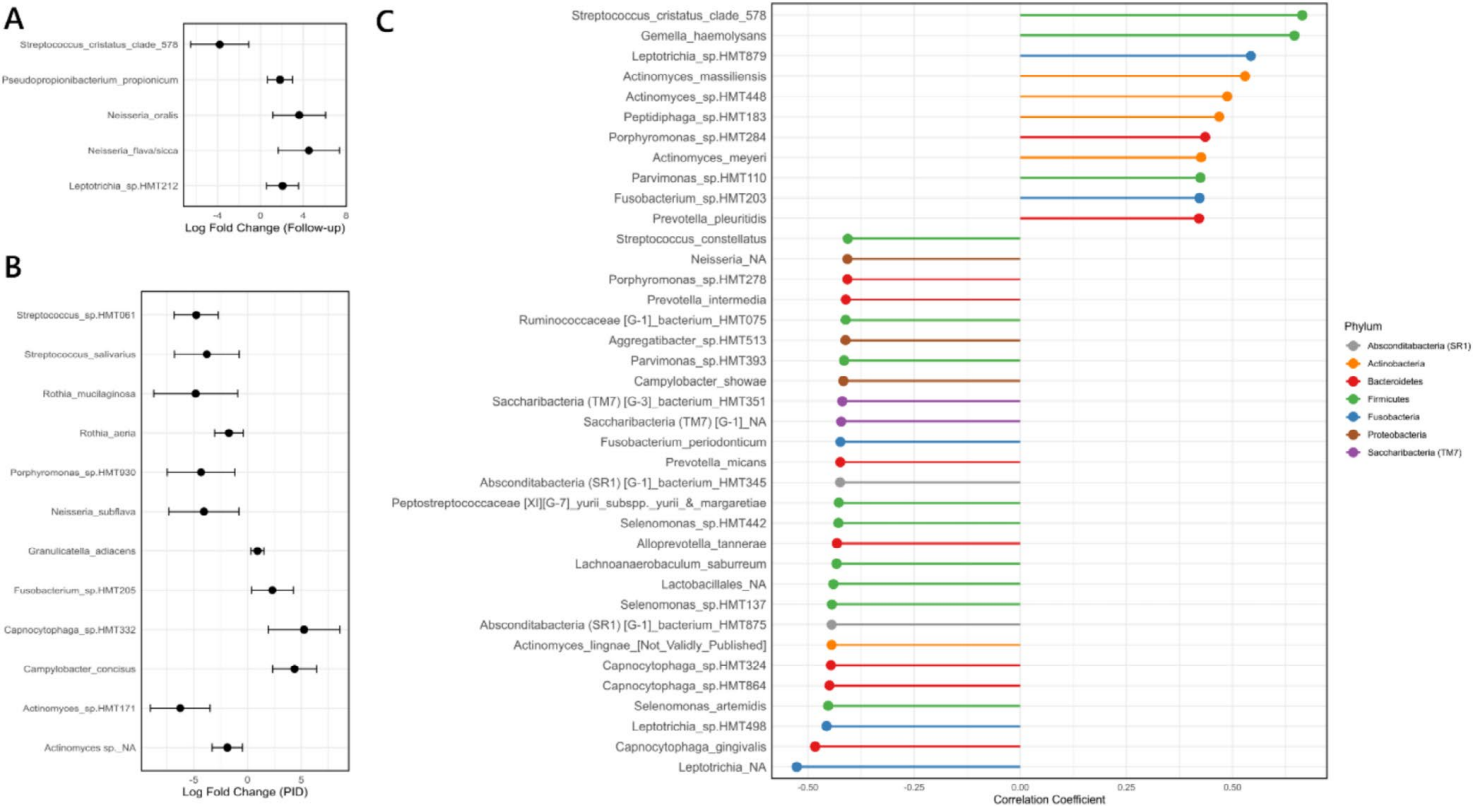
Differential abundance and correlation analysis of microbial taxa. (A) Differential abundance analysis of microbial taxa post therapy in PID children using the corncob method. The plot shows the log fold change in abundance of significant taxa between follow‐up and baseline. Error bars represent the 95% confidence intervals for the log fold change estimates. (B) Differential abundance analysis of microbial taxa at baseline between PID and non‐PID children using the corncob method. The plot shows the log fold change in abundance of significant taxa between different groups at baseline. Error bars represent the 95% confidence intervals for the log fold change estimates. (C) Correlation of CLR‐transformed abundance with absolute neutrophil counts for the PID children at baseline. The lollipop plot shows the correlation coefficients of significant taxa, coloured by phylum. Associations with coefficients > 0.4 and < −0.4 are shown.

On comparing the baseline subgingival microbiome of PID children and control children, PID children had a lower abundance of *Actinomyces* and *Neisseria* (Figure 3B). These taxa were notably observed to increase in the post‐therapy samples of PID children. Other taxa that were lower in abundance in PID children compared to control children included *Streptococcus salivarius, S. intermedius, Rothia aeria, R. mucilaginosa* and *Aggregatibacter* HMT 512 (Figures 3B and S3). Further, certain taxa such as *Campylobacter, Capnocytophaga, Leptotrichia shahii, Peptostreptococcus, P. micans, P*. HMT 300, *Selenomonas* and *Veillonellaceae* [G‐1] HMT 155 were more abundant in PID children compared to control children. Analysis using beta binomial models in corncob largely confirmed these findings (Figure 4). An increase in *Neisseria* spp. and a decrease in *Streptococcus cristatus* clade 578 were detected post therapy in PID children compared to their pre‐therapy samples (Figure 4A). Comparing baseline samples in PID children versus control children showed that health‐associated *Rothia* spp., *Actinomyces* spp. and *Streptococcus salivarius* were reduced in PID children. An increased abundance of *Campylobacter concisus* and *Capnocytophaga* sp. HMT 332 in PID children was detected relative to the control children (Figure 4B).

Several taxa were observed to correlate with absolute neutrophil counts for the pre‐therapy subgingival microbiome in PID children. These included taxa that were differentially abundant in PID children compared to control children at baseline, such as *Prevotella* spp., *Actinomyces* spp., *Capnocytophaga* spp. and *Saccharibacteria* spp. Strong positive associations could be observed between ANC and *Streptococcus cristatus* clade 578 and *Gemella haemolysans* (Figure 4C). Notably, these two taxa were observed to decrease in abundance post therapy (Figure 3A).

## 4 Discussion

Periodontitis, though uncommon in children (0.1%–1% prevalence), occurs at a notably higher rate with distinct microbiological profiles in children with PIDs, particularly those with congenital defects in phagocyte number or function. This study adds to the limited literature on periodontitis in this vulnerable group, offering a detailed evaluation of the subgingival microbiome in PID children compared to healthy controls. In our study, periodontitis affected 29% of the PID children, with no cases in controls, highlighting the increased susceptibility of PID‐affected children. These findings are consistent with Halai et al. (2020), who reported a periodontitis prevalence range of 33%–95% in similar populations.

### 4.1 Microbiome Diversity and Community Structure

Although the PID cohort is diagnostically diverse, including FA, SCN, AN, PLS and PN, the clinical data indicate that, despite differences in aetiology and additional treatments (e.g., G‐CSF, azithromycin and prior dental interventions), the microbiomes at baseline were broadly similar based on alpha/beta diversity metrics and the rarefied species abundances (Figures 1, 2 and S2). Nearly all patients, except two in the gingivitis subgroup, exhibited significant reductions in microbial alpha diversity following therapy, suggesting that while microbial richness and evenness decreased, specific taxa remained dominant. PERMANOVA analysis confirmed significant shifts in community structure (Bray‐Curtis and Jaccard indices), reflecting a microbial profile closer to that of healthy controls, a hallmark of successful periodontal intervention, as noted in prior studies linking reduced microbial diversity with improved periodontal health (Johnston et al. 2021; Kistler et al. 2013; Nath et al. 2022). Although larger subtype‐specific studies are needed, our data suggest that the immunological deficit (neutropenia) and the subgingival niche may provide a shared biological basis for the microbial shifts observed after periodontal treatment. Baseline alpha diversity in PID children was lower than control, a trend that persisted post therapy. This is consistent with literature suggesting that compromised immune function in PID patients can lead to a less diverse microbiome (Al‐Nesf et al. 2021; Y. Wang et al. 2021). However, non‐significant changes in some diversity measures (e.g., Shannon, Simpson and observed richness) suggest that factors beyond immune status could influence microbial diversity.

### 4.2 Differential Abundance of Microbial Taxa

The study identified specific microbial taxa with differential abundance before and after therapy in PID patients. Health‐associated taxa such as *Actinomyces* spp. and *Neisseria* spp. increased post therapy, while pathogenic taxa such as *Porphyromonas* spp. and *Prevotella* spp. significantly decreased (Figure 3). Compared to controls, baseline samples from PID children showed a lower abundance of beneficial and well‐documented nitrate‐reducing taxa such as *Actinomyces*, *Rothia* and *Neisseria*, which increased post therapy (Doel et al. 2005). This suggests periodontal therapy reduced disease‐associated microbes and promoted beneficial taxa, potentially restoring a more balanced microbial community, consistent with the goals of treatment in reducing pathogenic load and promoting a beneficial microbial profile (Teles et al. 2006).

### 4.3 Correlation With Neutrophil Counts

The correlation between certain taxa and ANC before therapy provides insights into the immune system's role in shaping periodontal microbial ecology. Strong positive associations with taxa such as *Streptococcus cristatus* clade 578 and *Gemella haemolysans* , which decreased post therapy, suggest that these microbes thrive when neutrophil abundance is relatively preserved. Conversely, taxa that correlate negatively with ANC, such as *Leptotrichia, Capnocytophaga, Selenomonas, Saccharibacteria* and *Prevotella*, may thrive under reduced neutrophil abundance (Lehman and Segal 2020).

*Streptococcus cristatus* and *Gemella haemolysans* are typical commensals in the oral cavity, and their reduction post therapy, despite a positive association with ANC, may indicate that they proliferate in response to subclinical inflammation or tissue damage where neutrophils maintain homeostasis rather than contribute to pathology. *S. cristatus* can inhibit the growth of *Porphyromonas gingivalis* , a key periodontal pathogen (Ho et al. 2017; B. Wang et al. 2009). The decrease in *S. cristatus* post therapy likely reflects a reduction in the overall bacterial load and inflammatory signals that attract neutrophils. Similarly, *G. haemolysans* , generally non‐pathogenic but found in higher abundances in certain oral diseases, likely reflects successful microbial modulation post treatment, reducing the inflammatory milieu supporting its proliferation (Miyoshi et al. 2021; Wade 2013). In contrast, taxa correlating negatively with ANC, such as *Leptotrichia, Capnocytophaga, Selenomonas, Saccharibacteria* and *Prevotella*, are typically associated with periodontal disease and inflammation. These organisms thrive in anaerobic environments caused by the characteristic tissue damage and immune dysfunction of periodontal disease. Their reduction post therapy highlights the effectiveness of non‐invasive clinical interventions in improving host–microbial ecological relationships. This study is the first to identify such putative taxa potentially regulated by neutrophil abundance within the periodontium.

### 4.4 Clinical Relevance and Implications

The significant prevalence of periodontal diseases in PID patients highlights the need for consistent oral health monitoring and timely interventions. Evidence of bone loss highlights the potential for lasting dental and systemic complications if left untreated (Nibali et al. 2021). Although follow‐up assessments showed clinical improvements, full periodontal health restoration was rare, emphasising the chronic nature of periodontal disease in PID patients. This suggests that ongoing maintenance and possibly adjunctive therapies are needed for optimal outcomes.

This study adds to the growing evidence on the microbiome's role in periodontal health, particularly in immunocompromised populations. Previous research has shown that PID patients have dysbiotic gut and skin microbiomes, characterised by altered diversity and increased pathogenic taxa (Oh et al. 2013; Pellicciotta et al. 2019; Sokol et al. 2019). We observed similar patterns in the oral microbiome and demonstrated that non‐surgical therapy can shift the subgingival microbiota towards a more health‐associated profile. Nevertheless, this study is subject to several limitations. The cohort included diverse primary immunodeficiencies, each characterised by unique underlying mechanisms and varying severity of neutropenia. While neutropenia served as a unifying characteristic across the cohort, this heterogeneity and the associated small overall sample size limit the generalisability of the findings to specific subtypes of PID. Additionally, differences in therapy exposure, such as the number of professional mechanical plaque removal (PMPR) visits, reflect the practical realities of clinical care in a heterogeneous population but pose challenges in standardising interventions, particularly for rare and complex conditions such as PIDs. Furthermore, the 6‐month follow‐up period may not fully capture long‐term trends in microbiome shifts or the sustainability of the clinical improvements observed. Correlations between neutrophil counts and microbial taxa were analysed for the entire PID cohort rather than separately for healthy and gingivitis/periodontitis patients due to sample size constraints. These highlight the need for future studies with larger stratified cohorts and extended follow‐up periods to better elucidate the interplay between PID, microbiome dynamics and periodontal therapy outcomes.

Although we found a correlation between ANC and specific microbial taxa, this relationship may differ in other neutrophil dysfunctions, such as LAD Type I, where neutrophils fail to migrate into tissues, causing severe disease from immunopathology rather than bacterial invasion alone (Moutsopoulos et al. 2015). Our findings should be viewed in the context of neutropenic conditions, where reduced neutrophil counts impair bacterial control in the periodontal environment. Future research should explore these variations across PIDs to better understand their impact on periodontal health. Despite these limitations, the strengths of this study include its robust methodological approach, high‐resolution sequencing and comprehensive bioinformatics analyses, which collectively provide novel insights into the interplay between neutropenia, the oral microbiome and periodontal therapy.

## 5 Conclusion

In conclusion, our study expands the understanding of how primary immunodeficiencies and the oral microbiome interact, highlighting the importance of considering both host and microbial factors in periodontal disease. Further research exploring the interactions between host immunity, microbial composition and clinical outcomes is essential for developing targeted and effective treatment strategies for children with PID.

## Author Contributions

N.D., L.N., J.D. and R.P.A. conceptualised the study and designed the research methodology. N.D. secured funding and supervised the project. Data collection and experimental work were performed by A.S.S., S.W., C.S. and L.N., with A.S.S. leading the laboratory analyses. Statistical analyses and data curation were conducted by A.S.S., S.W., C.S. and J.D. Data visualisation and figure preparation were handled by A.S.S., who along with S.W. wrote the manuscript draft. All authors reviewed and edited the manuscript, providing feedback and approval of the final version.

## Conflicts of Interest

The authors declare no conflicts of interest.

## Supporting information


**Data S1.** Supporting Information.

## Data Availability

The data that support the findings of this study are openly available in European Nucleotide Archive at https://www.ebi.ac.uk/ena/, reference number PRJEB76903.

## Appendix A

### DNA Extraction

Subgingival plaque samples were processed using the MasterPure Gram Positive DNA Purification Kit (Lucigen, USA). An overnight lysozyme incubation step was performed in the provided lysis buffer on a 1:1 ratio after thawing the samples, using the ReadyLyse lysozyme. The lysate was incubated in Proteinase K at 65°C for 30 min with mixing every 5 min. The protein in the samples was then precipitated and pelleted at 4°C and > 15,000*g*. DNA was then precipitated from the aqueous supernatant using isopropanol at a ratio of 12:7, and the pellet was rinsed with 75% v/v ethanol before resuspending in TE buffer and stored at −80°C until use.

### Sequence Processing

Adapter and primer sequences were removed using cutadapt (Martin 2011). Reads were quality‐filtered: forward and reverse reads trimmed to 270 and 240 bp, respectively. Trimmed reads with maximum expected errors (maxEE) > 2 (forward) or > 4 (reverse) were discarded. Maximum number of ambiguous bases (maxN) was set to 0. Filtered reads were used to model read error profiles, then denoised and merged. Chimeric sequences were removed from merged reads. Amplicon sequence sariants (ASVs) were assigned taxonomies using the eHOMD train set (version 15.1) (F. Escapa et al. 2020). Sequence counts of ASVs mapped to Human Microbial Taxa (HMT) up to genus, species or sub‐species levels were agglomerated in *phyloseq* (McMurdie and Holmes 2013).

### Statistical and Diversity Analyses

Aggregated microbial sequence counts in the samples were rarefied for 1000 iterations at a read depth of 5200 in the R package vegan. Statistical testing was performed on the rarefied beta diversity using permutational multivariate analysis of variance (PERMANOVA) in the R package *adonis*, with significance reported as the proportion of all rarefied iterations where *p* < 0.05 (Warton et al. 2012). Statistical testing of rarefied alpha diversity metrics from longitudinal samples was performed using linear mixed effects in the R package *nlme*, with significance reported as the proportion of all rarefied iterations that yielded *p* < 0.05 (Pinheiro et al. 2023). Alpha diversity between control and PID groups were tested using the Kruskal–Wallis test with post hoc pairwise comparisons using Wilcoxon signed rank tests with Bonferroni correction. Beta diversity between control and PID groups was tested using PERMANOVA in *adonis*. Differential abundance of taxa was analysed using analysis of composition of microbiomes with bias correction (ANCOMBC) and beta‐binomial models as implemented in the R package *corncob* (Lin et al. 2022; Martin et al. 2020). *p*‐values were adjusted for multiple comparisons using the Holm method to control the false discovery rate (FDR). The *corncob* package was used to assess the effect of covariates on both the abundance and dispersion of taxa, with correlation analysis of blood absolute neutrophil counts and aggregated microbial data conducted similarly.
