# Supplementary material for: Non‐Surgical Periodontal Therapy Modulates Oral Microbiome in Primary Immunodeficient Children
Source: J Clin Periodontol. 2025 Jul 20;52(10):1410–8. doi: 10.1111/jcpe.14201 (PMC12420080; doi:10.1111/jcpe.14201)
Supplement: Supplementary file 1 — Data S1. Supporting Information. [file JCPE-52-1410-s001.docx]

| Diagnosis of PID | Number  (n=24) | Neutrophil Disorder Category |
| --- | --- | --- |
| Severe Congenital Neutropenia Type 1 | 3 | Congenital Neutropenia. |
| SCN3, Severe Congenital Neutropenia Type 3  (Kostmann Syndrome) | 1 | Congenital Neutropenia. |
| Severe Congenital Neutropenia Type 4 | 1 | Congenital Neutropenia. |
| Chronic Idiopathic Neutropenia | 6 | Congenital Neutropenia. |
| Shwachman-Diamond Syndrome | 2 | Congenital Neutropenia. |
| Autoimmune Neutropenia | 4 | Congenital Neutropenia. |
| Clericuzio-type Poikiloderma with Neutropenia Syndrome | 1 | Congenital Neutropenia. |
| Glycogen Storage Disease Type 1b | 1 | Congenital Neutropenia. |
| Cohen Syndrome | 2 | Congenital Neutropenia. |
| Fanconi’s Anaemia | 2 | Bone Marrow Failure |
| Papillon LeFevre Syndrome | 1 | Defects in Motility |

**Table 1.** Diagnoses of PID participants.

| Characteristic | PID Group (n=24) | Healthy Controls (n=24) |
| --- | --- | --- |
| Male: Female | 13:11 | 15:9 |
| Mean Age (years) | 9.67 ± 3.70 | 10.04 ± 3.51 |
| G-CSF Treatment | 6 | None |
| G-CSF Treatment + Azithromycin | 3 | None |
| Further Antibiotics | 4 | None |
| Additional Medical Complications | 7 | None |
| Patients Using Inhalers | None | 2 |
| PMPR prior to study participation | 8 | None |
| Orthodontic Treatment | 4 | 1 |
| Dental Extractions | 3 | None |
| Fillings | 2 | 5 |
| Early Tooth Loss  (not trauma related) | 2 | 2 |
| History of Ulceration | 18 | 4 |
| Periodontal Disease at baseline | 7 | 0 |
| Periodontal Disease at follow-up | 4 | N/A |
| Gingivitis at baseline | 8 | 4 |
| Gingivitis at follow-up | 6 | N/A |
| Gingival health at baseline | 9 | 20 |
| Gingival health at follow-up | 10 | N/A |

Table 2. Summary of patient characteristics and intra-oral findings

| Characteristic | PID Treatment Group (n=9) | PID Diagnosis Key |
| --- | --- | --- |
| Male: Female | 6:3 | KS: Kotsmann Syndrome  PN: Persistent Neutropenia (Unknown genetics)  SCN: Severe Congenital Neutropenia  FA: Fanconi’s Anaemia  AN: Autoimmune Neutropenia  PLS: Papillon-Lefèvre Syndrome |
| Mean Age (years) | 9.78 ± 2.35 |  |
| G-CSF Treatment | 2 |  |
| G-CSF Treatment + Azithromycin | 1 |  |
| Further Antibiotics | 3 |  |
| PMPR prior to study participation | 3 |  |
| Dental Extractions | 3 |  |
| Early Tooth Loss  (not trauma related) | 2 |  |
| History of Ulceration | 6 |  |
| Periodontal Disease at baseline | 4 (KS, PN, SCN, FA) |  |
| Periodontal Disease at follow-up | 0 |  |
| Gingivitis at baseline | 3 (SCN, AN) |  |
| Gingivitis at follow-up | 1 |  |
| Gingival health at baseline | 2 (PLS, AN) |  |

Table 3. Summary of patient characteristics from Figure 1, whose follow-up samples were analysed.


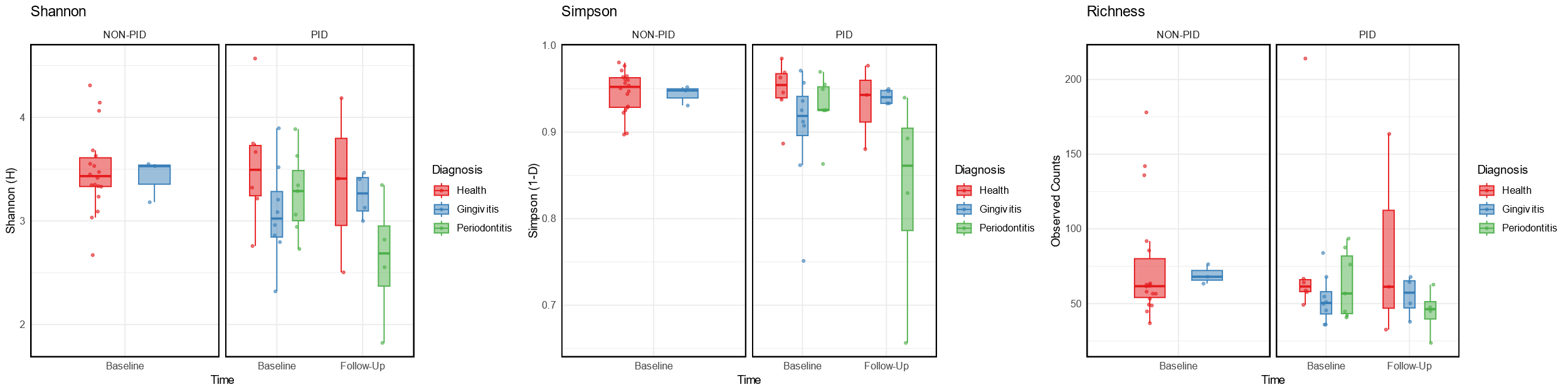


Figure S1. Alpha diversity measures (Shannon, Simpson, and Observed richness) stratified by diagnosis groups (Health, Gingivitis, and Periodontitis) within PID and NON-PID samples. Each panel shows the distribution of alpha diversity metrics for baseline and follow-up samples, grouped by PID (Primary Immunodeficiency) and NON-PID (Non-Immunodeficient) categories. Jittered points represent individual sample values, while the boxplots show the median and interquartile ranges for each group.


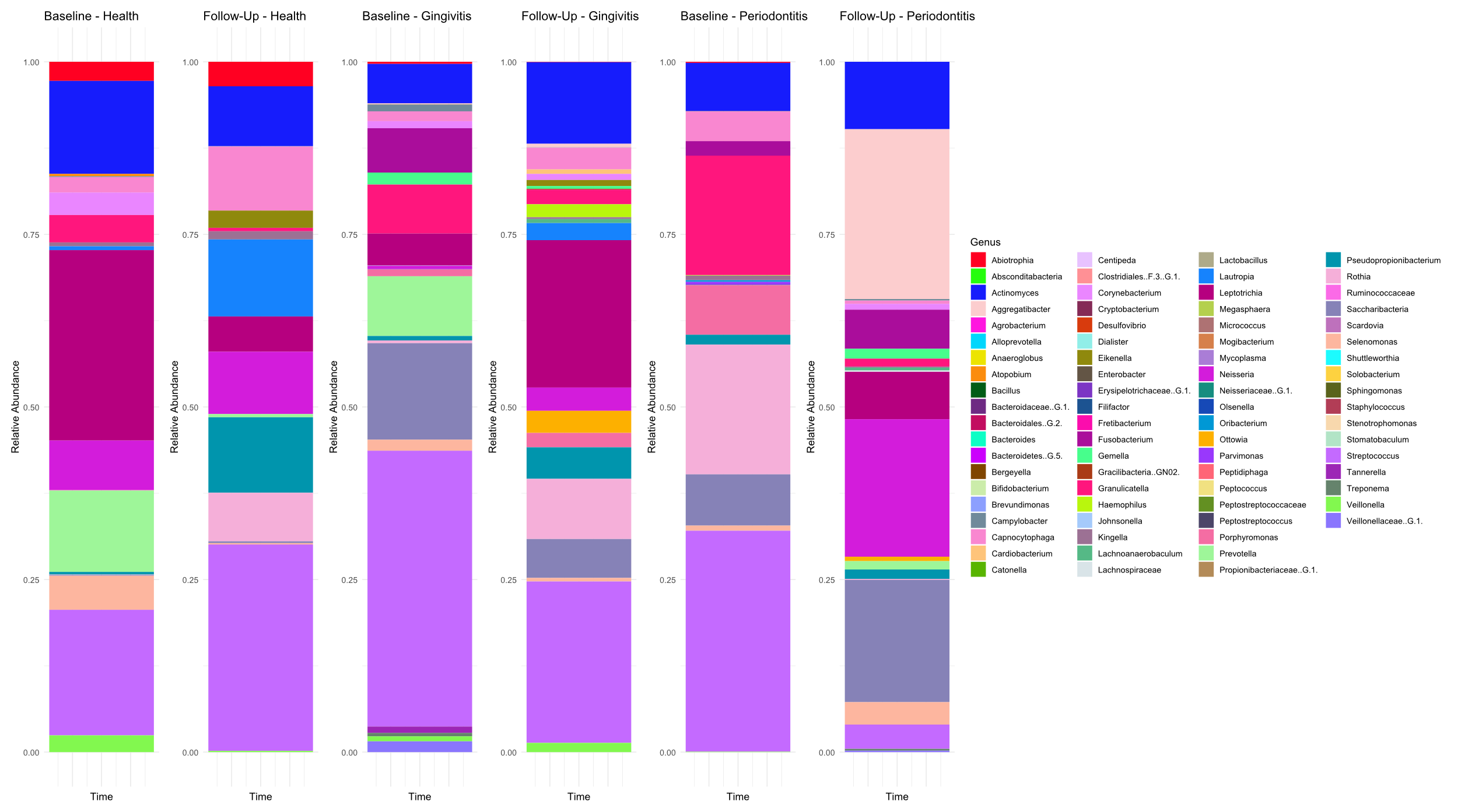


Figure S2. Stacked bar plots representing the relative abundance of various genera in the microbiome samples. The plots display rarefied data from the baseline and follow-up stages for three clinical diagnoses: Health, Gingivitis, and Periodontitis. For each diagnosis group, the genus-level taxonomic composition is visualized, highlighting changes over time between baseline and follow-up.


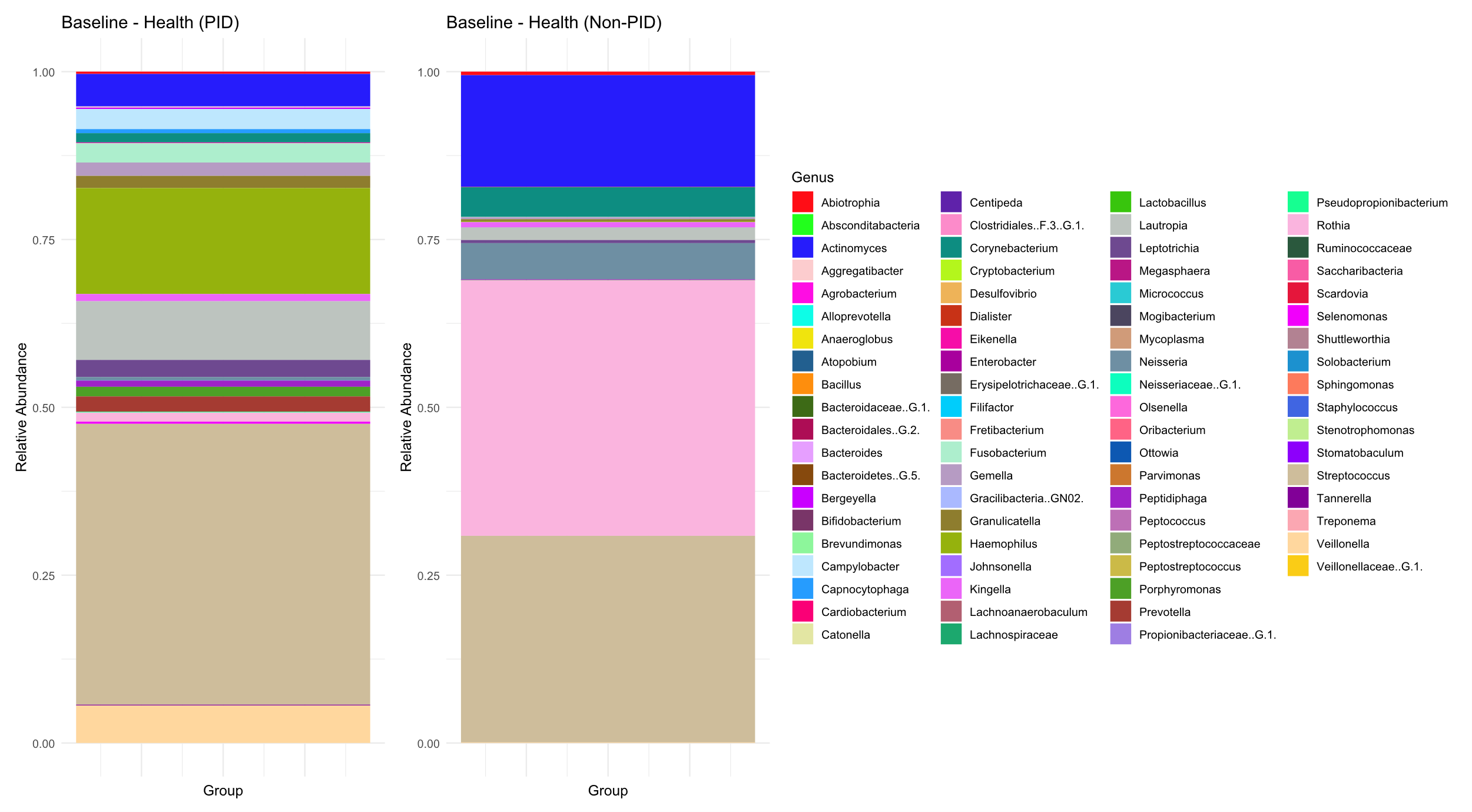


Figure S3. Stacked bar plots illustrating the relative abundance of rarefied genera in the microbiome samples for PID and Non-PID groups diagnosed as healthy.
